# Clearance of Human IgG1-Sensitised Red Blood Cells *In Vivo* in Humans Relates to the *In Vitro* Properties of Antibodies from Alternative Cell Lines

**DOI:** 10.1371/journal.pone.0109463

**Published:** 2014-10-10

**Authors:** Kathryn L. Armour, Cheryl S. Smith, Natasha C. Y. Ip, Cara J. Ellison, Christopher M. Kirton, Anthony M. Wilkes, Lorna M. Williamson, Michael R. Clark

**Affiliations:** 1 Department of Pathology, University of Cambridge, Cambridge, United Kingdom; 2 Bristol Institute for Transfusion Sciences, Bristol, United Kingdom; 3 National Health Service Blood and Transplant, Cambridge, United Kingdom; 4 Department of Haematology, University of Cambridge, Cambridge, United Kingdom; San Raffaele Scientific Institute, Italy

## Abstract

We previously produced a recombinant version of the human anti-RhD antibody Fog-1 in the rat myeloma cell line, YB2/0. When human, autologous RhD-positive red blood cells (RBC) were sensitised with this IgG1 antibody and re-injected, they were cleared much more rapidly from the circulation than had been seen earlier with the original human-mouse heterohybridoma-produced Fog-1. Since the IgG have the same amino acid sequence, this disparity is likely to be due to alternative glycosylation that results from the rat and mouse cell lines. By comparing the *in vitro* properties of YB2/0-produced Fog-1 IgG1 and the same antibody produced in the mouse myeloma cell line NS0, we now have a unique opportunity to pinpoint the cause of the difference in ability to clear RBC *in vivo*. Using transfected cell lines that express single human FcγR, we showed that IgG1 made in YB2/0 and NS0 cell lines bound equally well to receptors of the FcγRI and FcγRII classes but that the YB2/0 antibody was superior in FcγRIII binding. When measuring complexed IgG binding, the difference was 45-fold for FcγRIIIa 158F, 20-fold for FcγRIIIa 158V and approximately 40-fold for FcγRIIIb. The dissimilarity was greater at 100-fold in monomeric IgG binding assays with FcγRIIIa. When used to sensitise RBC, the YB2/0 IgG1 generated 100-fold greater human NK cell antibody-dependent cell-mediated cytotoxicity and had a 10^3^-fold advantage over the NS0 antibody in activating NK cells, as detected by CD54 levels. In assays of monocyte activation and macrophage adherence/phagocytosis, where FcγRI plays major roles, RBC sensitised with the two antibodies produced much more similar results. Thus, the alternative glycosylation profiles of the Fog-1 antibodies affect only FcγRIII binding and FcγRIII-mediated functions. Relating this to the *in vivo* studies confirms the importance of FcγRIII in RBC clearance.

## Introduction

For 40 years, human polyclonal anti-RhD antibodies have been used successfully in the prophylactic treatment of haemolytic disease of the foetus and newborn to prevent the immunisation of RhD-negative women by RhD-positive foetal RBC. The precise mechanisms by which the polyclonal anti-RhD IgG suppress immunisation against the RhD antigen are not fully understood but involve rapid, non-inflammatory, FcγR-mediated sequestration of the RhD-positive cells [1], [2]. There is evidence that FcγRIIIa plays the major role in this clearance of sensitised RBC. Most notably, RBC clearance was slower following administration of an anti-FcγRIII monoclonal antibody to chimpanzees and to a patient [3], [4]. Due to the problems implicit in the use of antibodies from hyperimmune plasma, there has been a drive to identify effective monoclonal anti-RhD antibodies with which to replace polyclonal anti-RhD. As a result, monoclonal anti-RhD antibodies form perhaps the largest group of different antibodies against the same antigen that have been tested in humans. It appears that the most efficient antibodies for RBC clearance are those that give good antibody-dependent cell-mediated cytotoxicity (ADCC) with NK cells [5], [6]. This does not necessarily imply that NK cells are involved in RBC clearance but that this assay is a good measure of ability to interact with FcγRIIIa. Phagocytosis by splenic macrophages is held to be the mechanism of IgG-sensitised RBC destruction but to achieve this by engagement of the high affinity IgG receptor, FcγRI, would require displacement of serum IgG, which occupies its binding site under physiological conditions. Strong binding of RBC-bound antibody to the intermediate affinity FcγRIIIa may allow rapid association of RBC and macrophages. This could both activate the macrophages directly and promote interactions via FcγRI molecules upon dissociation of non-specific IgG from their binding sites.

One of our interests lies in the development of mutated human IgG constant regions with different combinations of properties that can be tailored for therapeutic use. Combining these constant regions with the variable regions of the human anti-RhD IgG1 antibody Fog-1 [7] allowed measurement of their activity in various *in vitro* assays and offered the potential to study their effect on the intravascular survival of RBC in humans. Accordingly, aliquots of autologous RBC were labeled with different radionuclides and coated with either Fog-1 IgG1 antibody or a mutated version with reduced effector function (Fog-1 G1Δnab) before reinjection [8]. As anticipated, clearance of cells coated with Fog-1 G1Δnab from the circulation was significantly slower than the clearance of wild-type IgG1-coated cells. IgG1-mediated clearance was complete and irreversible, with accumulation in the spleen and liver and the appearance of radiolabel in plasma. Notably, the clearance mediated by our recombinant Fog-1 IgG1 was much more rapid than seen in a previous study that used the original Fog-1 antibody at comparable coating levels [9]. Monoclonal anti-RhD IgG do range widely in their ability to mediate RBC clearance and, whilst some of this variation results from the properties of the different variable regions and the choice of IgG1 or IgG3 constant regions, the cell line used for expression of the IgG appears to be crucial [5]. It is therefore relevant that the original Fog-1 was obtained from human-mouse heterohybridoma cells following fusion of Epstein-Barr virus-transformed B lymphocytes with the mouse myeloma line X63-Ag8.653 [10] whereas transfected YB2/0 rat myeloma cells were used for the production of both recombinant Fog-1 G1 and G1Δnab. The cell line influences the effector properties of an antibody sample by being responsible for its glycosylation profile.

IgG heavy chain carbohydrate moieties are linked to N297 of each chain, fill the space between the two CH2 domains and play roles in the stability and interactions of the Fc (reviewed [11]). Each oligosaccharide is of the complex biantennary type and consists of a basic heptasaccharide structure that can be enlarged by the presence of fucose on the primary N-acetylglucosamine (GlcNAc) residue, galactose (±sialic acid) on one or both of the terminal GlcNAc and a bisecting GlcNAc residue. Absence of carbohydrate results in a decrease in binding to all classes of Fc receptor whilst changing the oligosaccharide structure can modify binding. Serial truncation of Fc carbohydrate structures results in the movement of CH2 domains towards each other and conformational changes in the FcγR interface region that make receptor binding less favourable [12].

Apart from the Fog-1 studies, the only clinical investigation of anti-RhD antibodies that were produced from alternative cell lines involved a BRAD-5 and BRAD-3 mixture [13]. In contrast to the dissimilar RBC clearance rates of the two Fog-1 IgG1, comparable rates were mediated by the BRAD antibodies from EBV-immortalised human cell lines and CHO cells. We wished to understand the difference in potency between the Fog-1 IgG1from YB2/0 and the original Fog-1 from human-mouse heterohybridoma cells by comparing the properties of IgG carrying glycosylation that is typical of the products of rat or mouse cell lines. We chose to compare the YB2/0-produced Fog-1 IgG1 with the same antibody produced in the mouse myeloma cell line NS0 since this is a line commonly used for therapeutic antibody production.

Comparison has previously been made between the activities of antibodies produced in YB2/0, NS0 and CHO cells [14]. When produced in YB2/0 cells, the humanised IgG1 antibody CAMPATH-1H was approximately 30-fold more efficient in ADCC assays than the same antibody made in either NS0 or CHO cells whilst the antibodies were equally active in monocyte killing assays. Oligosaccharide analysis showed that YB2/0-produced antibody contained less fucose (6–7% against 90% for CHO) and more bisecting GlcNAc residues than IgG made in CHO or NS0. These two properties are related since fucosylation prevents enzymatic addition of bisecting GlcNAc. The NS0- and CHO-derived antibodies contained carbohydrate of a similar structure but the NS0 IgG1 was significantly underglycosylated. Fractionation of the IgG showed that it was the lack of fucose, rather than the presence of bisecting GlcNAc, that led to YB2/0-produced IgG1 being more efficient at ADCC [15]. Furthermore, when YB2/0 cells were caused to over-express FUT8 mRNA to supplement their low levels α1,6-fucosyltransferase, this led to IgG1 with 81% fucosylation and 100-fold lower ADCC.

Since most of the literature that examines the effects of cell-specific glycosylation involves IgG produced in CHO cells, there has been no systematic comparison of YB2/0- and NS0-produced IgG. Although NS0 and CHO cell lines yield IgG with similar distributions of glycan structures, the presence of aglycosyl IgG in NS0 samples potentially reduces binding to all classes of FcγR. Here we evaluate the relative levels of binding of YB2/0- and NS0-produced Fog-1 IgG1 to a series of transfected cell lines that separately bear each type of human FcγR. We also test the antibodies' performance in ADCC, NK cell and monocyte activation and macrophage adherence and phagocytosis assays. So that the impact of altering the glycosylation profile of the antibody can be compared to the change in binding achieved through amino acid mutation, in some assays we include the Fog-1 IgG1 mutant with reduced activity, Fog-1 G1Δnab, made in YB2/0 or NS0 cells. As well as definitively assessing the relative levels of interaction of YB2/0- and NS0-produced IgG1 with the different FcγR, this work reinforces what is known about the mechanism of IgG-sensitised RBC clearance.

## Materials and Methods

### Antibody production and characterisation

The production of recombinant IgG1 and mutant G1Δab forms of Fog-1 in YB2/0 rat myeloma cells [16] and their subsequent characterisation has been described [17]–[19]. Fog-1 G1Δnab was produced by removing the G1m(1,17) allotypic residues from G1Δab, without effect on its properties [8]. Both wildtype and mutant Fog-1 antibodies were similarly produced from NS0 mouse myeloma cells [20]. The antibodies are denoted as Fog-1 G1 and Fog-1 G1Δnab followed by (YB2/0) or (NS0). The relative concentrations of the antibodies were confirmed by sandwich ELISA, using goat anti-human IgG, Fc-specific antibodies and HRPO-conjugated goat anti-human κ light chains antibodies (Sigma, Poole, UK).

### Measurement of IgG binding to transfected cell lines bearing human FcγR

Cell lines transfected with appropriate cDNA expression vector constructs to express single human FcγR have been variously obtained. For FcγRI, the cell line was B2KA (S. Gorman and G. Hale, unpublished) and CHO cells expressing FcγRIIIb of allotypes NA1 and NA2 [21] were provided by J. Bux. CHO cell lines expressing FcγRIIIa of allotypes 158F and 158V as GPI-anchored receptors or the various FcγRII molecules as transmembrane proteins with native cytoplasmic domains have been constructed [22], [23]. Continued and uniform expression of the appropriate FcγR was confirmed in each antibody binding assay by staining a sample of the cells for the receptor. Cells were incubated with CD64 (clone 10.1), CD32 (AT10) or CD16 (LNK16) monoclonal antibody (AbD Serotec, Kidlington, UK) and its binding detected with FITC-conjugated goat anti-mouse IgG antibodies (Sigma).

Binding of monomeric Fog-1 IgG to B2KA cells expressing FcγRI was measured as previously described using Fog-1 IgG2 antibody, produced in YB2/0, as a negative control [17]. Monomeric binding of IgG to FcγRIIIa was detected by the same protocol but using biotinylated goat F(ab′)_2_ anti-human κ (Rockland) followed by ExtrAvidin FITC (Sigma). Complexed Fog-1 antibody binding to FcγRII and III receptors was measured by pre-incubating the test antibodies with equimolar amounts of goat F(ab′)_2_ fragments that recognise human κ chain (Rockland) [19]. Human IgA1, κ purified myeloma protein (The Binding Site, Birmingham, UK) was used as a negative control test antibody. Complexes were detected using FITC-conjugated F(ab′)_2_ fragments of rabbit anti-goat IgG, F(ab′)_2_-specific antibodies (Jackson ImmunoResearch, Newmarket, UK) or FITC-conjugated donkey anti-goat IgG antibodies (Serotec). The mean fluorescence of cells from each sample was determined using a CyAn ADP flow cytometer and Summit v4.3 software (DakoCytomation, Ely, UK) or on a FACScan flow cytometer using LysisII software (Becton Dickinson, Oxford, UK).

Fold-differences in IgG binding were calculated as follows: Firstly, a curve was fitted to a subset of the mean fluorescence data of G1 (YB2/0), the higher-binding IgG. This was a logarithmic curve for the complexed IgG binding or a sigmoidal curve for the monomeric IgG binding. Using the mean fluorescence values of each of the three highest concentrations of the other IgG, the ratio of concentrations of the two IgG giving these mean fluorescence values was calculated. This was carried out for three or more independent experiments so that the fold-difference in binding could be expressed as the mean±sd of at least nine values.

### Rosetting assays

O, RhD-positive RBC, which were shown to carry 9000 RhD sites/cell by SOL-ELISA [8], were incubated with dilutions of Fog-1 antibodies in V-bottom plates for 1 hour at room temperature. The cells were pelleted, washed three times in 150 µl/well PBS and resuspended in 100 µl RPMI (approximately 1% suspension). CHO+FcγRIIIa 158F or 158V cells were harvested using Cell Dissociation Buffer (Invitrogen), washed, resuspended at 4×10^6^ cells/ml and 100 µl samples added to the sensitised RBC. The cells were pelleted together at 200× g for 2 min and incubated on ice for 1 h. 10 µl samples were transferred to slides with coverslips and representative images captured at 40× magnification.

### Assays of functional responses to Fog-1 antibody-sensitised RBC

ADCC, macrophage adhesion and phagocytosis and monocyte activation assays were carried out using human cells as described previously [23]. NK cell activation was assessed using a method adapted from [24]. Peripheral blood mononuclear cells (PBMC) were prepared from FCGR3A- and FCGR2C-genotyped donors by adding 6 ml samples of EDTA-anti-coagulated blood to 45 ml samples of RBC lysis buffer (150 mM NH_4_Cl, 10 mM KHCO_3_, 1 mM EDTA) and incubating at room temperature for 15 min. White cells were collected by centrifugation, washed in RBC lysis buffer and resuspended in complete RPMI (RPMI containing 10% heat-inactivated FBS, 2 mM L-glutamine, 0.5 µg/ml amphotericin B, 100 U/ml penicillin, 0.1 mg/ml streptomycin). Using the same donor as for the rosetting assays above, RBC were prepared by pelleting cells from 100 µl blood, washing twice in 1 ml RPMI and resuspending in complete RPMI. Samples of test antibody, 10^5^ PBMC, 4×10^5^ RBC were added to round-bottomed wells in 100 µl complete RPMI in triplicate and incubated at 37C in a humidified atmosphere of 5% CO_2_ in air for 20 h. Control wells omitted test antibody or used 10 µg/ml IgG1 of irrelevant specificity. The surface expression of CD54 on NK cells (identified as CD3^−^CD56^+^) was determined by flow cytometry: The cells in each well were washed three times with FACS wash buffer (PBS containing 0.1% BSA, 0.1% NaN_3_) then incubated in 100 µl FACS wash buffer containing PerCP/Cy5.5-conjugated CD3 (clone UCHT1), PE-conjugated CD56 (clone HCD56) and APC-conjugated CD54 (clone HA58, all from BioLegend, London, UK) for 45 minutes on ice. The cells were washed twice and fixed in 1% formaldehyde. Samples were analysed using a CyAn ADP flow cytometer and Summit v4.3 software with appropriate compensation settings. Mean APC fluorescence intensity was calculated for at least 2000 CD3^−^CD56^+^ cells from each well and plotted as mean±SD of the triplicate samples at each test antibody concentration. Samples containing control IgG1 antibody or RhD negative RBC showed no increase in APC fluorescence relative to samples containing no test antibody.

### FCGR3A and FCGR2C genotyping

Typing for the FcγRIIIa 158F/V polymorphism was carried out on genomic DNA that had been purified using the QIAamp DNA Blood Mini Kit (Qiagen, Manchester, UK). A 235 bp section of DNA, which comprised parts of the 5th exon and following intron, was amplified using oligonucleotides (5′ CATATTTACAGAATGGCAAAGG 3′, 5′ CAACTCAACTTCCCAGTGTGAT 3′) that each mismatch the closely homologous FCGR3B gene at their 3′ nucleotide. The PCR products were directly sequenced using the second primer. Lack of FCGR3B contamination was confirmed by examining the electropherogram at two positions within the amplified DNA where the FCGR3A and FCGR3B sequences differ and the presence of the TTT (F) codon, GTT (V) codon or a mixture was determined.

High homology between the ectodomains of FcγRIIb and FcγRIIc means that FcγRIIc typing requires amplification of cDNA to enable use of a FcγRIIb/c-specific primer at the 5′ end and a FcγRIIa/c-specific primer at the 3′ end. Whole blood was diluted 10-fold with RBC lysis buffer and the white cells collected by centrifugation. These were lysed and stored in RNASafer (Omega bio-tek, Lutterworth,UK) before RNA was prepared using Tripure reagent (Roche, Burgess Hill, UK). First strand cDNA was synthesised from a FcγRIIa/c-specific primer (5′ AGCAAGTCTAGAGTATGACCACATGGCATAACGTTACTCTTTAG 3′) and was amplified by PCR using the same oligonucleotide in conjunction with a FcγRIIb/c-specific primer (5′ GACTGCTGTGCTCTGGGCGCCAGCTCGCTCCA 3′). Product was subjected to a second round of PCR using primers F (5′ AGGGAGTGATGGGAATCCTGTCATT 3′) and R (5′ CATAGTCATTGTTGGTTTCTTCAGG 3′). The nested PCR product was directly sequenced from primer F2 (5′ CATATGCTTCTGTGGACAGCT 3′). For this cDNA segment, the FcγRIIc ORF and STP alleles differ at 3 positions in addition to the CAG (Q)/TAG (STP) codon corresponding to amino acid residue 13 [25].

## Results

### Comparison of binding of YB2/0 and NS0-generated antibodies to human FcγR

We used transfected cell lines, each expressing a single human FcγR, to pinpoint the effects on FcγR binding of changing the IgG production cell line. For FcγRIIa, FcγRIIIa and FcγRIIIb, functional polymorphisms are known [26] and testing was performed for two allotypes of each receptor. The IgG were titrated to obtain receptor-binding curves from which any differences in strengths of binding could be calculated. Such comparisons are traditionally made by relating the concentrations of each antibody required to give half-maximal binding but, for the IgG concentrations used here, either maximal binding was not achieved by the higher-binding IgG or the lower-binding IgG did not reach the half-maximal binding level. Therefore, comparison was made between the binding signals detected for the highest three concentrations of the lower-binding IgG and the binding curve of the higher-binding IgG as described in Materials and Methods.

**Figure 1:**
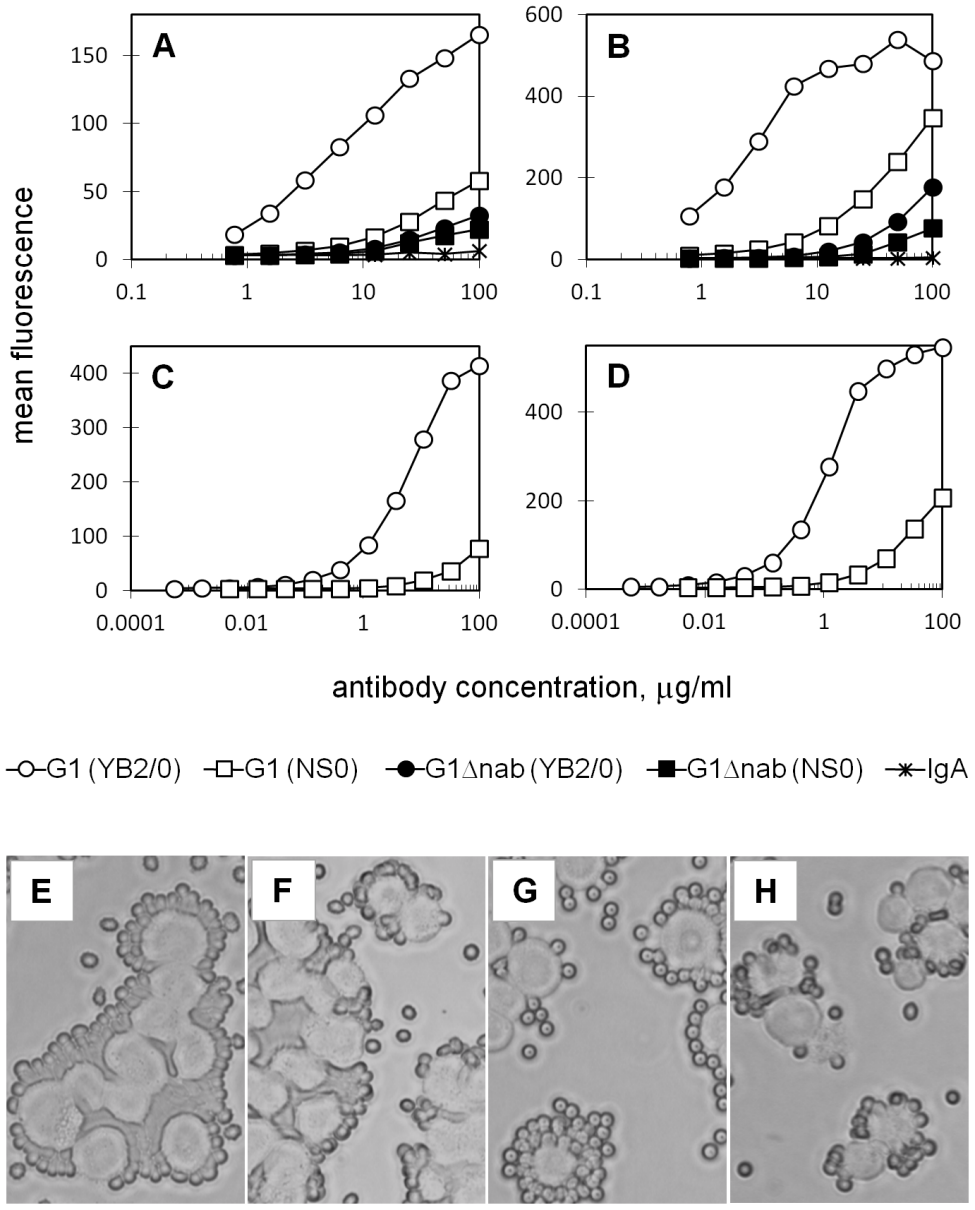
Binding interactions of YB2/0- and NS0-produced Fog-1 G1 and G1Δnab antibodies with human FcγRIIIa. **A–D** CHO cells expressing FcγRIIIa of allotypes 158F (A, C) and 158V (B, D) were incubated with pre-complexed (A, B) or monomeric (C, D) Fog-1 IgG and binding detected with fluorescent reagents and flow cytometry. Graphs show mean fluorescence of ≥12 000 cells at each antibody concentration and are typical of the results obtained in at least three experiments with each receptor. **E–H** Examples of the rosetting of FcγRIIIa 158V-expressing cells by RBC sensitised with Fog-1 G1 (YB2/0) at 10 µg/ml (E) and 1.1 µg/ml (F) or with Fog-1 G1 (NS0) at 100 µg/ml (G) and 11 µg/ml (H). Images are typical of eight independent experiments.

Given the previous reports of FcγRIIIa importance in RBC clearance, we began by examining binding to this receptor. The binding of pre-complexed Fog-1 G1 (YB2/0) antibody to cells expressing FcγRIIIa of allotype 158F was 45-fold higher than that of pre-complexed G1 (NS0) (45±17 fold over 5 experiments; Figure 1A). For the higher affinity allotype of the receptor, FcγRIIIa 158V, G1 (YB2/0) complexes bound 20-fold better than complexes of the NS0-produced antibody (20±5 fold over 3 experiments; Figure 1B). This assay of complexed IgG binding to FcγRIIIa is sufficiently sensitive for binding of the Fog-1 IgG1 mutant with reduced effector function (Fog-1 G1Δnab) to be detected at the highest IgG concentrations used. Binding of G1Δnab (YB2/0) to the FcγRIIIa 158F and 158V molecules is 51-fold and 63-fold lower, respectively, than that of G1 (YB2/0) (158F: 51±12 fold over 3 experiments; 158V: 63±18 fold over only 2 experiments). Switching to NS0 for G1Δnab antibody production only gives a small additional decrease in binding.

FcγRIIIa is classed as a medium affinity Fc receptor and its interactions with monomeric antibody samples can also be measured. This was carried out to ensure that superiority of the YB2/0-derived antibody was not an artefact of the complexed antibody assay system. By this method, G1 (YB2/0) bound 102-fold and 97-fold more efficiently than G1 (NSO) to the 158F and 158V allotypes of the receptor respectively (158F: 102±25 fold over 3 experiments; 158V: 97±33 fold over 3 experiments; Figure 1C and D). The higher affinity of the 158V allotype of the receptor is clearly evident here since the binding curves for this allotype are displaced towards lower concentrations compared to the curves for the F allotype.

As an additional binding measurement, Fog-1 G1-sensitised RBC were tested for their ability to rosette the receptor-bearing cells. Figure 1, panels E - H show photographs from a representative experiment carried out with the FcγRIIIa 158V cell line. IgG with Fog-1 variable regions are known to give 100% saturation of RBC RhD sites (equivalent to 9000 IgG/RBC in these experiments) at a coating concentration of approximately 20 µg/ml and 50% saturation at 0.4 µg/ml (data not shown). A reduction in G1 (NSO) coating concentration from 100 µg/ml to 11 µg/ml reduced rosette formation even though this does not represent a large change in terms of IgG/RBC. Tight rosettes, similar to those seen at100 µg/ml G1 (NSO), were formed by RBC coated with G1 (YB2/0) at 1.1 µg/ml or approximately 6000 IgG/RBC. The difference between the G1 (YB2/0) and G1 (NSO) coating concentrations that gave equivalent levels of rosetting was greater in the case of the FcγRIIIa 158F cell line (data not shown).

**Figure 2:**
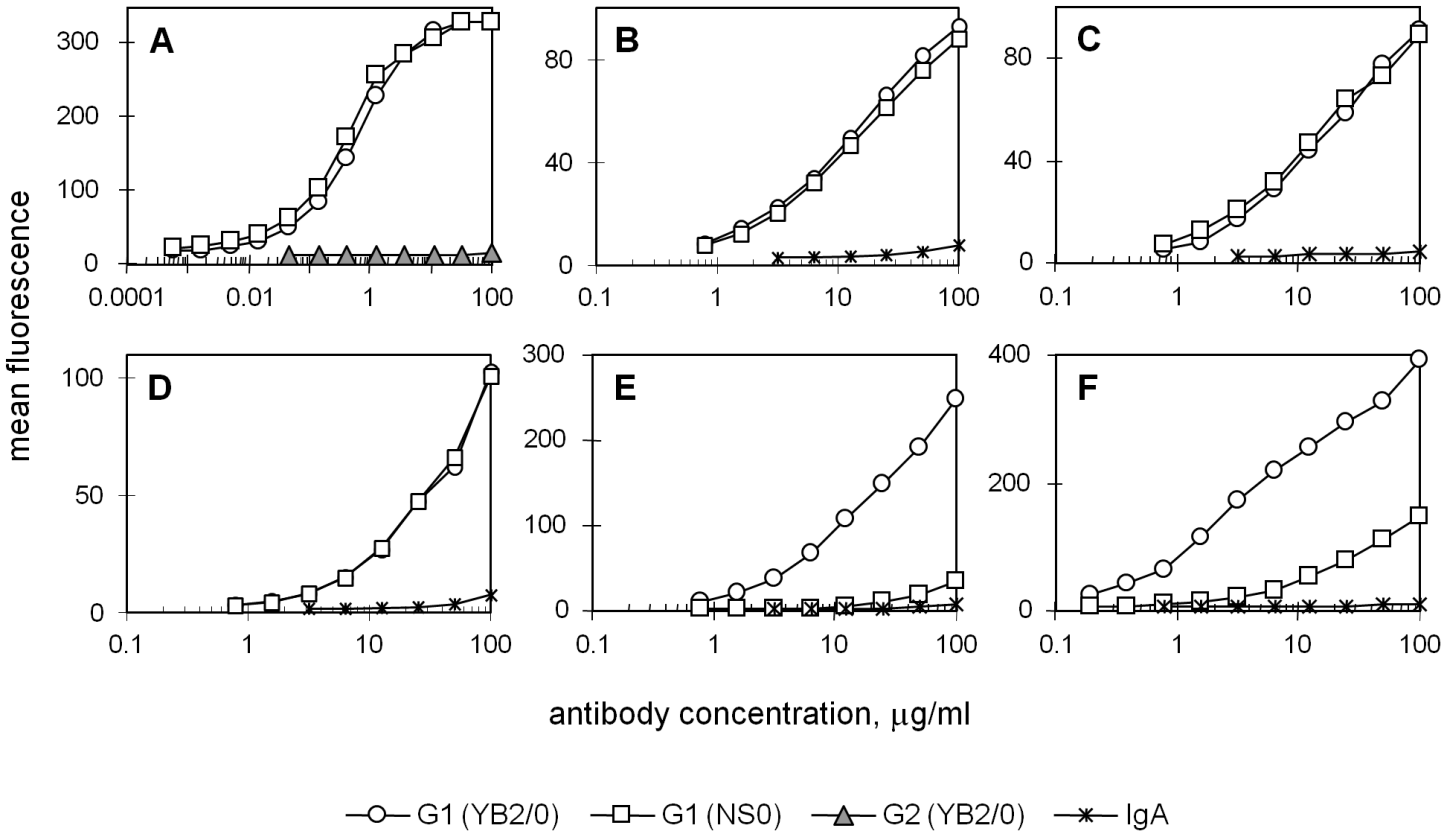
Binding of YB2/0- and NS0-produced Fog-1 G1 antibodies to human FcγR. **A** Binding of monomeric IgG was measured for FcγRI using the B2KA cell line and flow cytometry. Fog-1 IgG2 antibody, produced in the YB2/0 cell line, was used as the non-binding control antibody for this receptor. **B–F** Binding of pre-complexed IgG was measured using CHO cell lines expressing the low affinity receptors which were FcγRIIa, allotypes 131R (B) and 131H (C), FcγRIIb (D) and FcγRIIIb, allotypes NA1 (E) and NA2 (F). The level of background binding is given by the negative control antibody, IgA,κ. Graphs show mean fluorescence of ≥12 000 cells at each antibody concentration and are typical of the results obtained in at least three experiments with each receptor.

We went on to compare the binding of monomeric YB2/0- and NS0-produced immunoglobulin to the high affinity FcγRI and complexed antibody binding to the remaining human FcγR, which are of low affinity. Very similar binding of the two IgG1 antibodies was observed for FcγRI, FcγRIIa (of allotypes 131R and 131H) and FcγRIIb (Figure 2, panels A–D). In contrast, for FcγRIIIb of NA1 and NA2 allotypes (Figure 2E and F), there were large differences in binding with the YB2/0-produced IgG1 being 39-fold and 38-fold better respectively (NA1: 39±12 fold over 3 experiments; NA2: 38±10 fold over 3 experiments). These differences are comparable to those measured for the FcγRIIIa molecules.

### Measurement of functional cellular responses to Fog-1-sensitised RBC

**Figure 3:**
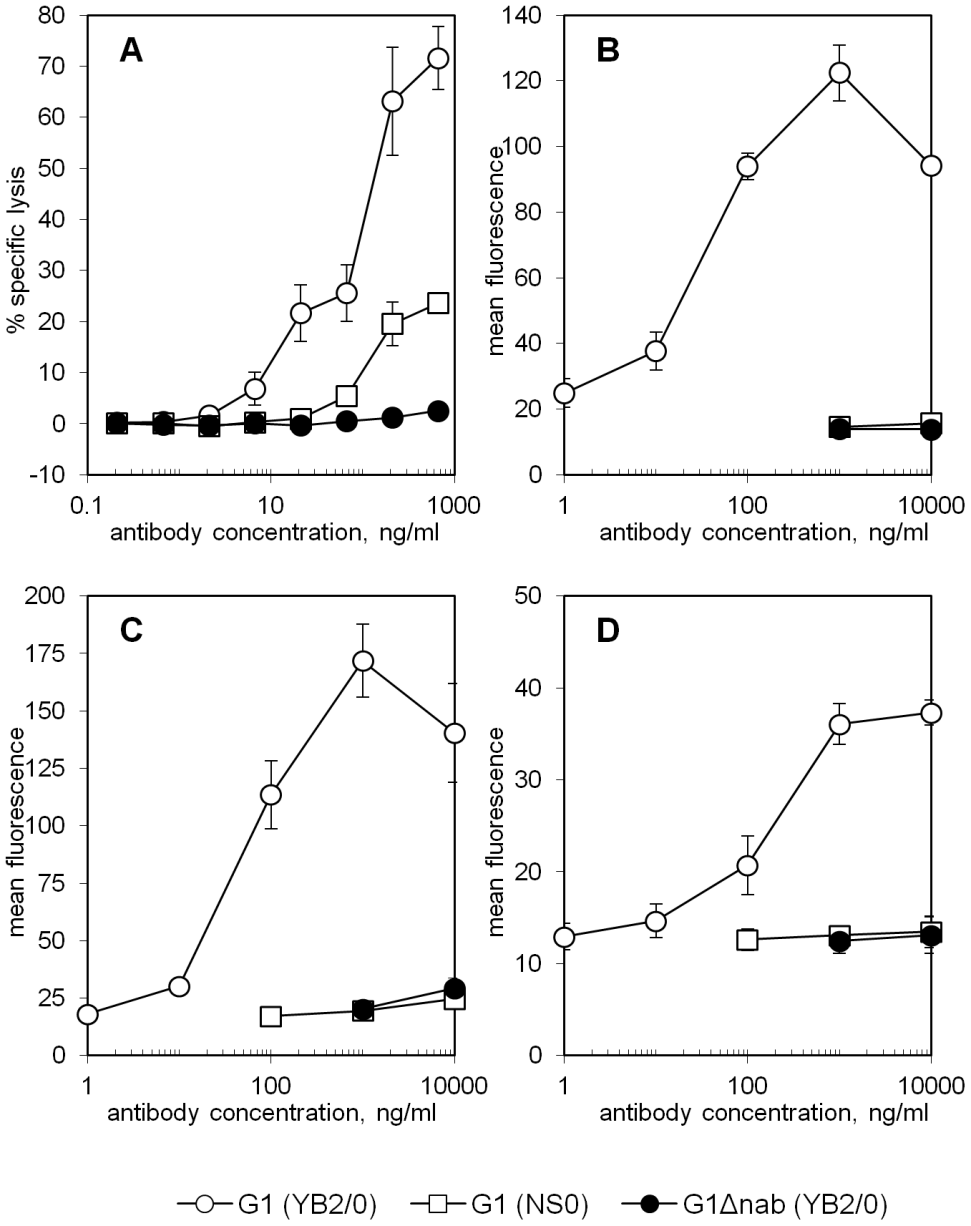
Functional responses of NK cells to RBC sensitised with Fog-1 antibodies. **A** The specific lysis of sensitised RBC by NK-cell mediated ADCC is presented as mean±SD of triplicate samples. This experiment used effector cells pooled from 6 donors but similar results were obtained in four experiments with individual donors of PBMCs. **B–D** The activation of NK cells in response to sensitised RBC as visualised by the level of CD54 on the surface of CD3^−^, CD56^+^ lymphocytes. Each graph shows the mean±SD of triplicate samples for each data point. Donors of PBMC were of the following genotypes: FcγRIIIa 158V/V, FcγRIIc 13Q/13Q (B), FcγRIIIa 158F/V, FcγRIIc 13STP/13STP (C) and FcγRIIIa 158F/F, FcγRIIc 13STP/13STP (D). CD54 signals for samples with no test antibody were 14.2±0.1 (B), 17.0±2.5 (C) and 13.0±1.1 (D). CD54 signals for samples incubated with irrelevant IgG1 were 13.3±0.4 (B), 18.7±4.1 (C) and 12.1±0.9 (D).

Measurement of NK cell-mediated ADCC of Fog-1 IgG-sensitised RBC showed G1 (YB2/0) to cause lysis about 100-fold more efficiently than G1 (NS0) at sub-saturating concentrations (Figure 3A). However, the lysis mediated by G1 (NS0) was still higher than the background levels of lysis that were typical of G1Δnab (YB2/0).

We also examined the interaction with NK cells by using flow cytometry to measure the levels of the NK cell activation marker CD54 after overnight co-culture of PBMC and RBC in the presence of Fog-1 antibody. Results are shown for three donors of PBMC with different FCGR3A/FCGR2C genotypes (Figure 3, panels B–D). G1 (YB2/0) concentrations ≥1 ng/ml (Figure 3B), ≥10 ng/ml (Figure 3C) or ≥100 ng/ml (Figure 3D) produced CD54 levels that were significantly higher than in samples with no test antibody (p<0.05, Student's *t*-test). Even at 10 µg/ml, G1 (NS0) caused little increase in CD54 level with the fluorescence signals being similar to those seen in response to G1Δnab or non-specific IgG1 control and in samples without test antibody. Since 10 µg/ml G1 (NS0) generated a CD54 level that was similar to or lower than 10 ng/ml G1 (YB2/0), the two IgG appear to be at least 1000-fold different in their abilities to activate NK cells.

**Figure 4:**
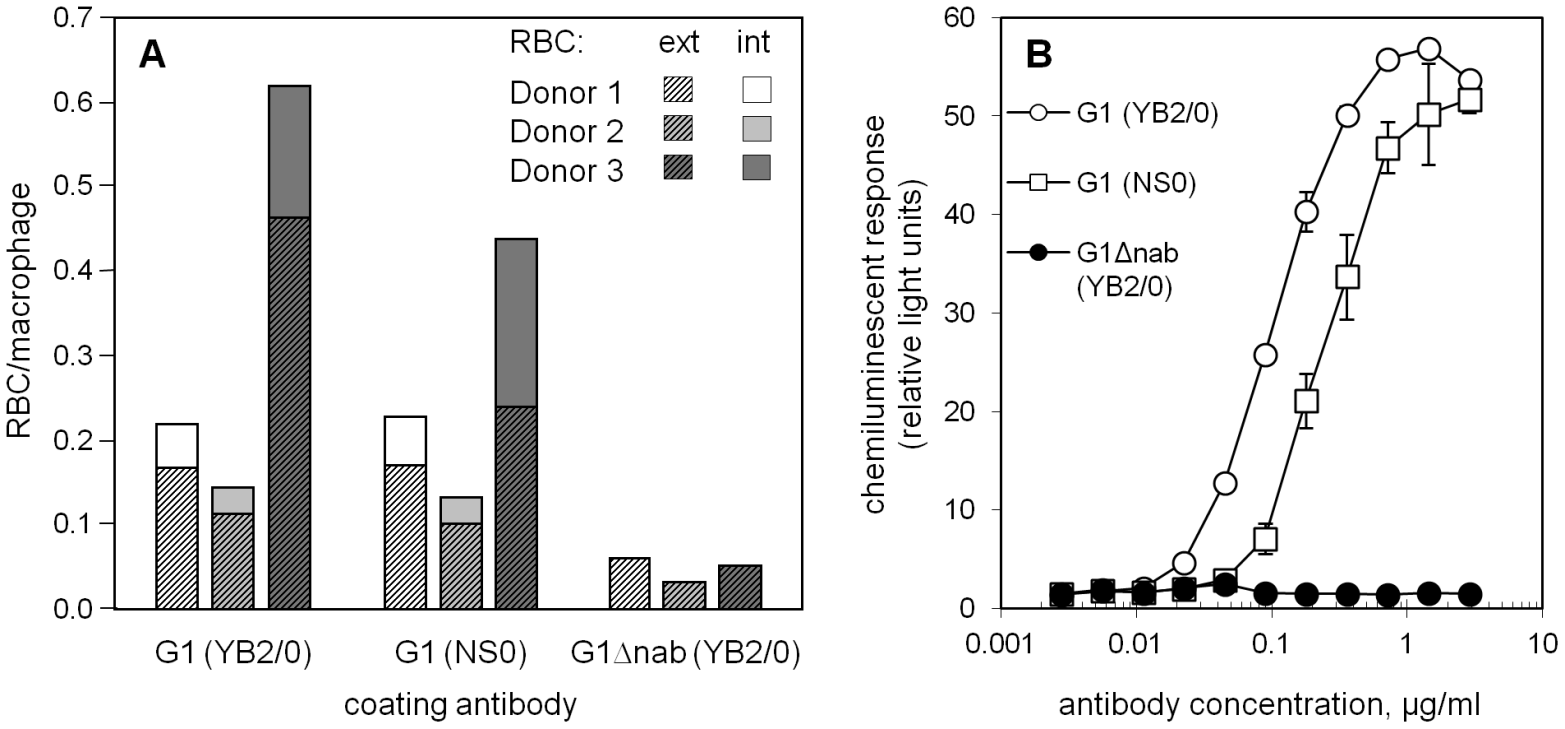
Interactions of Fog-1 IgG-sensitised RBC with monocytes and macrophages. **A** The numbers of adherent (ext) and phagocytosed (int) RBC per macrophage were determined for RBC sensitised with saturating concentrations of Fog-1 IgG. Results are shown for macrophages from three different donors. The numbers of unsensitised RBC associating with macrophages were typically 15- to 20-fold lower than the numbers of Fog-1 G1 (YB2/0)-sensitised RBC. **B** The mean chemiluminescent response of monocytes to sensitised RBC is plotted with the error bars indicating the range of the duplicate samples.

The activities of G1 (YB2/0) and G1 (NS0) were also compared using macrophages and monocytes as effector cells. Both of these cell types express FcγRI in addition to lower affinity receptors. Human macrophages were obtained from adherent mononuclear cells that had been cultured with M-CSF and differentiated with IFNγ and LPS and were CD64^+^, CD32^+^ and CD16^+^ by flow cytometry (data not shown). The proportions of macrophages found to be interacting with Fog-1 antibody-saturated RBC following 1 h incubations were determined. For three different macrophage donors, there was no significant difference between the G1 (YB2/0) and G1 (NS0) samples in terms of the numbers of RBC interacting (p = 0.4, paired Student's *t*-test) or the proportion of these that had been phagocytosed (p = 0.3) (Figure 4A). Presence of the G1 antibodies resulted in higher macrophage/RBC interaction rates than G1Δnab and there were no instances of phagocytosis with the latter antibody.

Chemiluminescence assays of monocyte activation in response to Fog-1 antibody-sensitised RBC showed Fog-1 G1 (YB2/0) to be approximately 3-fold more efficient at activating monocytes than G1 (NS0) (Figure 4B). G1Δnab did not cause activation.

## Discussion

Our comparison of the activities of IgG1 antibodies produced in YB2/0 and NS0 cell lines has shown heightened performance of the YB2/0 antibody in FcγRIII binding and FcγRIII-mediated functions. With the amino acid sequence of the two IgG1 antibodies being identical, the difference in activity must be due to variations in glycosylation. Although we did not analyse the glycosylation profiles of the IgG tested here, previous studies have shown that NSO-produced antibody was underglycosylated compared to IgG produced in YB2/0, their glycan moieties contain more fucose and less bisecting GlcNAc residues [14] and that lower levels of fucose lead to greater efficiency in ADCC [15].

We were able to pinpoint through which receptors the differences in activity were generated by comparing the binding of YB2/0- and NS0-produced Fog-1 G1 to each type of human FcγR. The assays measured monomeric IgG binding to the high affinity FcγRI and medium affinity FcγRIIIa and complexed IgG binding to FcγRII and FcγRIII molecules. For each of the receptors FcγRI, FcγRIIa 131R and131H and FcγRIIb, G1 (YB2/0) and G1 (NS0) showed very similar binding to each other. In contrast, large inequalities in binding ability between G1 (YB2/0) and G1 (NS0) were observed in complexed IgG binding to both FcγRIIIa and FcγRIIIb and in monomeric IgG binding to FcγRIIIa. In addition, Fog-1 G1 (YB2/0)-sensitised RBC readily formed rosettes with the FcγRIIIa transfectants at lower IgG coating concentrations than Fog-1 G1 (NS0)-sensitised RBC.

The superior FcγRIIIa binding of G1 (YB2/0) over G1 (NS0) translates into greater NK cell-mediated ADCC activity as might be expected since it is the only Fc receptor carried by NK cells in the majority of people. In some individuals, NK cells also express FcγRIIc, a low affinity, activating receptor with identical extracellular domains to FcγRIIb. This is due to a polymorphism, corresponding to amino acid 13 of the mature protein, where functional FcγRIIc depends on the presence of a Q codon, rather than stop codon [27]. In addition to the ADCC assays, we compared the abilities of the antibodies to cause NK cell activation when incubated with PBMC and RhD-positive RBC. Fog-1 G1 (YB2/0) was much more efficient in increasing levels of the activation marker CD54 than the NS0 antibody. Although our study was limited to one individual of each *FCGR3A* 158 genotype, with only the VV donor being FcγRIIc-positive, we did see a trend in the lowest G1 (YB2/0) concentration at which significant activation was detected: VV<FV<FF. This is consistent with the activation being dependent on FcγRIIIa avidity for IgG.

In contrast to the large differences in activity seen in ADCC and NK cell activation experiments, G1 (YB2/0)- and G1 (NS0)-coated RBC interacted with macrophages to a similar extent and there was only a three-fold variation in ability to activate monocytes. In these two assays, the high affinity FcγRI, which binds the two antibodies equally efficiently, is present and able to play a dominant role in the absence of high levels of competing non-specific IgG. This is unlikely to be the case *in vivo* where the concentrations of IgG mean that FcγRI molecules will be occupied by antibody and macrophage FcγRIIIa becomes more important for RBC removal.

The difference in FcγRIIIa binding of IgG1 from YB2/0 and NS0 cell lines is similar to that between YB2/0- and CHO-derived IgG1 with the former being approximately 100-fold better at binding to soluble FcγRIIIa 158 F or V by ELISA [28]. A variant CHO line, Lec13, produces IgG molecules with 10% fucosylation and dimers of these IgG show up to 50-fold improved binding over normal CHO-produced IgG to FcγRIIIa α-chain in ELISAs [29]. Lower fucose gave less improvement when measuring the binding of trimers or mutated IgG with an intrinsically higher affinity for FcγRIIIa. This concurs with the results of our three measurements of FcγRIIIa binding where the greatest difference was observed when monomeric IgG was tested. These groups [28], [29] showed essentially identical binding of their glycosylation variants to FcγRI and FcγRIIa, allotype 131H although the low fucose antibodies gave slightly higher signals with FcγRIIa, allotype 131R and FcγRIIb. The difference in ADCC efficacy between G1 (YB2/0) and G1 (NS0) is also similar to that observed between YB2/0- and CHO-derived IgG1 in several studies. In ADCC assays using 20 different donors of PBMC, a YB2/0-produced version of the CD20 therapeutic antibody, rituximab, was 10–100 fold better than the original CHO-produced antibody [28]. YB2/0–produced IgG gave equivalent levels of ADCC to CHO-made antibody at lower antigen densities [30], at lower antibody concentrations or with lower numbers of effector cells [31].

The effect of switching from CHO- to YB2/0-produced IgG1 on the interaction with FcγRIIIa has also been analysed by surface plasmon resonance and isothermal titration calorimetry [32]. The enhancement of affinity arises mainly from an increased association rate that is a consequence of greater favourable enthalpy and implies additional non-covalent interactions. In line with the approximate 100-fold difference we saw between monomeric YB2/0- and NS0-produced IgG1binding to cell-surface FcγRIIIa, low-fucose IgG1was shown to have a 50-fold greater affinity than high-fucose IgG1 for soluble FcγRIIIa 158V by surface plasmon resonance [33]. Mutation of the receptor residue N162 reduced the affinity of low-fucose IgG1 by 13-fold whilst increasing that of high-fucose IgG1 by 3-fold. The authors infer that a high affinity interaction requires glycosylation at N162 of the receptor but that this carbohydrate can only have productive contacts with nonfucosylated IgG. The fucose residue, which protrudes from the carbohydrate core, may prevent close approach of the molecules. Although the crystal structure of an IgG1-Fc fragment in complex with FcγRIIIb was solved using aglycosyl receptor [34], it does indicate that a carbohydrate moiety attached to receptor residue N162 would be orientated towards the carbohydrate of the Fc. Only FcγRIIIa and FcγRIIIb of human FcγR have a glycosylation site at position162, which accounts for the selectivity of the fucose effect.

Features of Fog-1 G1 (YB2/0)-mediated RBC clearance *in vivo*, namely the accumulation of RBC in the liver and the presence of radioactivity in the plasma [8], have raised concern about the use of YB2/0-produced IgG in the prevention of alloimmunisation [5]. The hepatic uptake could be indicative of YB2/0-derived antibodies having pro-inflammatory interactions via their carbohydrate residues with molecules of the innate immune system. Accumulation in the liver, in addition to the spleen, occurred in two subjects with>11000 IgG1 molecules/RBC (or more than 40 µg IgG1/ml packed cells) but not in a subject with 6800 molecules/cell. This accumulation must be FcγR-dependent since it did not occur in two subjects where RBC were coated with Fog-1 G1Δnab at>15000 molecules/cell. Few RBC clearance studies have included imaging but an investigation reported hepatic uptake at high coating levels when RBC were sensitised with different amounts of polyclonal anti-Rh antibody from human serum [35]. Thus accumulation in the liver may be a consequence of FcγR binding of IgG that is presented at a high density on the RBC surface rather than interactions specifically with the glycan structure of the YB2/0-produced IgG. Most RBC survival studies use low coating levels as they are designed to discover if relatively low, prophylactic doses of the anti-RhD IgG would give sufficient RBC clearance. This might also explain the unusualness of detecting radioactivity in the plasma, which has been suggested to imply that some RBC were destroyed by a potentially pro-inflammatory extracellular cytotoxic mechanism rather than by phagocytosis [5]. The fate of radioisotopes following phagocytosis is unknown and cannot be assumed to be the same as for other products of cell destruction such as haemoglobin. The high rates of RBC destruction in our study may account for the levels of radiolabel in the plasma. These peaked at 4–6% injected dose 100 min post-injection, by which time more than 95% of the injected doses had disappeared from the cell fractions [8]. Mollison et al. [35] saw comparable levels of plasma radioactivity where there were rapid rates of destruction but most studies have not reported plasma radioactivities. In addition to Fog-1 IgG1, two other YB2/0-produced anti-RhD antibodies have been tested in humans. R297 was found to be at least as effective as commercially-available polyclonal anti-RhD in clearing RBC [36]. Its derivative, R593 (roledumab), is undergoing clinical trials [37] and has been shown to be well tolerated and have a similar pharmacokinetic profile to human polyclonal anti-RhD [38].

Superior ADCC activity, as afforded by YB2/0 production, can be achieved by alternative methods of controlling the level of antibody fucosylation (reviewed [39]). Ideally, all carbohydrate moieties within the therapeutic antibody sample should be without fucose since fucosylated molecules in mixtures can complete for antigenic sites on target cells and batch-to-batch variation in carbohydrate composition is a regulatory issue. A FUT8^−/−^ CHO line that produces completely non-fucosylated antibodies but retains the growth characteristics of parent is attractive to the biopharmaceutical industry [40]. In ADCC, FUT8^−/−^ CHO-produced chimeric anti-CD20 IgG1 was 100-fold more efficient than original rituximab and 2–3 fold better than YB2/0-produced antibody and enhancement was also seen for the other IgG subclasses [41]. As well as being an effective method of improving activity, removal of fucose should not result in immunogenicity since 10–20% of normal human IgG lacks fucose [42]. Clinical trials have shown that non-fucosylated antibodies are tolerated and can give clinical effects at low doses [43], [44].

This work has capitalised on a unique opportunity to compare the effects of alternative glycosylation profiles on the *in vivo* and *in vitro* properties of IgG molecules with the same amino acid sequence. Our comparison of YB2/0- and NS0-produced IgG1 antibodies has revealed that differences in Fc receptor binding are confined to FcγRIII and amount to 100-fold higher binding of YB2/0-produced IgG1 in the case of monomeric IgG binding to FcγRIIIa. The previously-reported underglycosylation of IgG from NS0 cells [14] would be expected to reduce binding to all FcγR but our results showed very similar binding to receptors of the FcγRI and FcγRII classes. The greater ability of Fog-1 IgG1 from YB2/0 cells, compared to the original Fog-1 from human-mouse heterohybridoma cells, to clear RBC *in vivo* therefore results from its improved FcγRIII binding. These pieces of evidence confirm the importance of FcγRIII in RBC clearance.
